# LYEU-Net: A lightweight yet efficient U-Net with multibranch feature aggregation for pavement crack segmentation

**DOI:** 10.1371/journal.pone.0356870

**Published:** 2026-08-25

**Authors:** Bing Yang, Jiaying He, Yuncheng Shen

**Affiliations:** College of Physics and Information Engineering, Zhaotong University, Zhaotong, China; The Hong Kong Polytechnic University, HONG KONG

## Abstract

Accurate segmentation of pavement cracks is crucial for intelligent transportation systems. However, a critical research gap exists in current methodologies: high-performance models require substantial computational resources, which renders them unsuitable for edge deployment, whereas existing lightweight models often sacrifice segmentation accuracy for low resource use. To address this challenge, we propose LYEU-Net, a lightweight yet efficient U-Net architecture that is designed to balance accuracy and efficiency. The model incorporates three key innovations: a feature extraction module (FEM) for improved salient feature extraction using skip connections, a multibranch feature extraction module (MBM) for efficient multiscale contextual feature capture, and a feature aggregation module (FAM) for adaptive feature weighting to refine decoder outputs. Experimental results across four diverse datasets (Crack500, ShadowCrack, GAPs384, and AigleRN-TRIMM) show that LYEU-Net achieves the best mIoU among the compared methods with only 0.134M parameters and 0.347G FLOPs. It improves precision and mIoU over existing methods, although slight decreases in recall or F1 score on some datasets indicate a precision–recall trade-off. This work provides a viable solution for real-time crack detection on mobile devices. The code and data are available at https://github.com/yangbing668/LYEU-Net.

## Introduction

Cracks are a common type of structural damage to infrastructure, such as buildings and roads. They not only impair aesthetics but also weaken load-bearing performance and accelerate pavement deterioration [1]. Traditional crack detection methods, which rely on manual inspection and subjective assessment, are time-consuming, labor-intensive, and inefficient [2]. Conventional techniques such as median filtering, thresholding algorithms, and edge detection often fail to achieve the accuracy and efficiency that are required for crack identification. Therefore, developing automated and accurate crack detection methods is crucial for ensuring structural safety and meeting routine road inspection demands.

Crack detection and segmentation remain complex and time-consuming tasks because of multiple influencing factors; thus, automated solutions are needed to increase operational efficiency. However, existing methods face a critical trade-off between accuracy and efficiency. High-performance models [3,4] often depend on complex architectures and large parameter sizes, which limits their deployment on resource-constrained mobile or edge devices. Although lightweight models [5,6] reduce computational costs, they often simplify feature extraction operations and may therefore weaken the ability to capture multiscale contextual information. This limitation is particularly evident in pavement crack segmentation because cracks usually exhibit irregular shapes, variable widths, low contrast, uneven grayscale distributions, and cluttered backgrounds under different imaging conditions [7]. Consequently, designing a lightweight model that can preserve strong multiscale feature representation remains a key challenge for practical crack segmentation.

Convolutional neural networks (CNNs), critical modules for image feature extraction, enable the automated extraction of key information and deliver exceptional performance without manual intervention. The advent of U-Net [8] has inspired numerous derivative models, including UNet++ [9], MEFA-Unet [10], Ret-UNet [11], and BC-TSEA-UNet [12]. In semantic segmentation tasks, classification on the basis of local features alone is prone to ambiguity. The incorporation of global context features can effectively improve the result; however, owing to the shortcomings of convolution kernels in terms of understanding the overall structure of the image and associating different features, the task of crack segmentation is still quite challenging [13]. In contrast, the transformer [14] and vision transformer (ViT) [15] architectures demonstrate superior performance in modeling long-range contextual dependencies, thus offering new perspectives for crack segmentation [16]. Nevertheless, large transformer-based models typically involve substantial numbers of parameters, which hinders their deployment on resource-constrained mobile devices.

**Fig 1:**
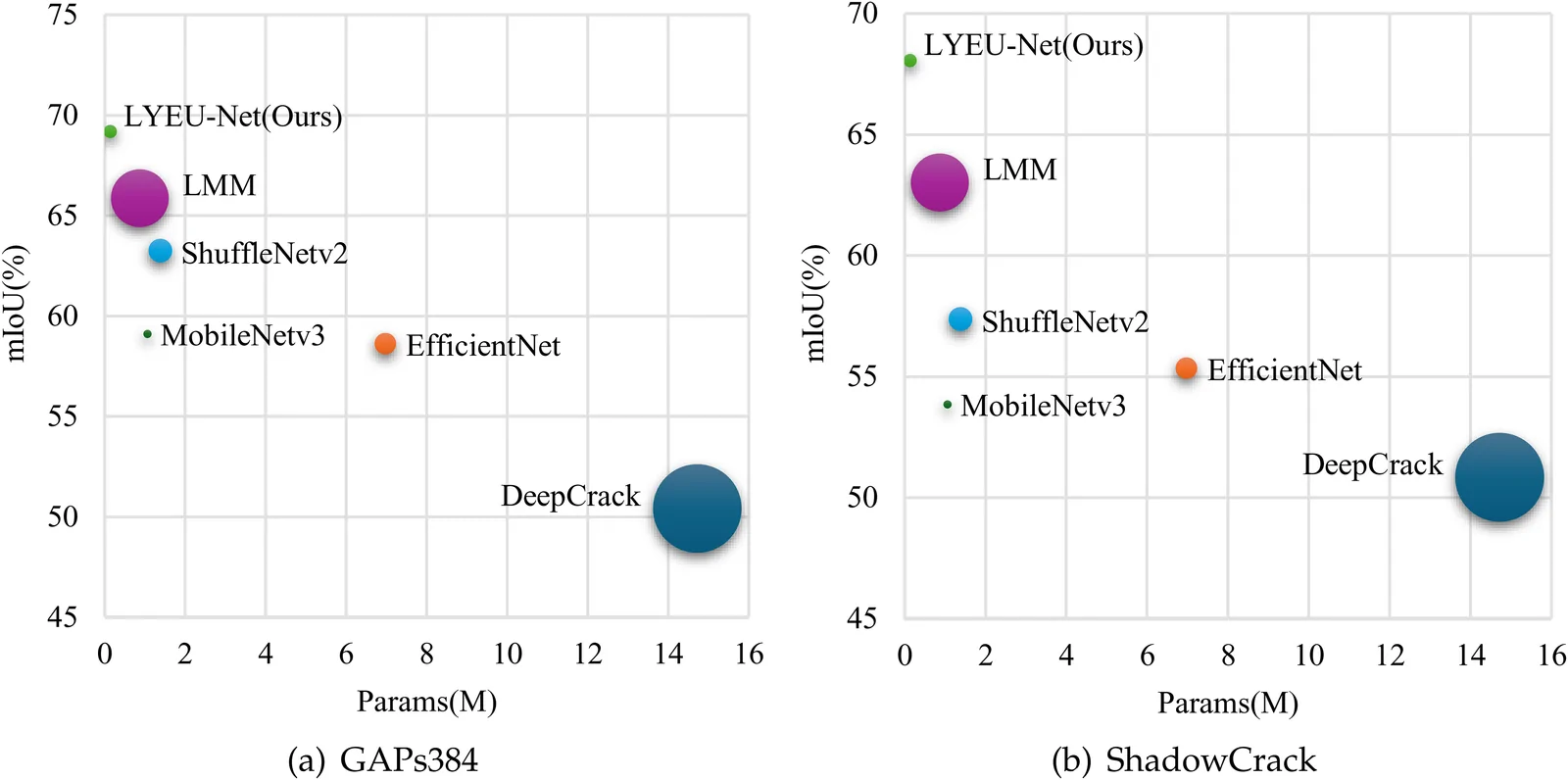
Evaluation of the Params (M), FLOPs (G) and mIoU (%) of the proposed model in the test dataset.

Lightweight crack segmentation has recently garnered significant attention as a critical yet challenging component of automated crack detection [17]. However, additional improvements can be made in terms of model complexity and performance. The comparative complexity and mean intersection over union (MIoU) of the proposed model are shown in Fig 1. Subfigures (a) and (b) present the visualization results in the GAPs384 and ShadowCrack datasets, respectively, with the x-axis denoting the model parameters (M), the y-axis representing the mIoU (%), and the bubble sizes corresponding to the FLOPs (G).

To address this gap, we propose LYEU-Net, a lightweight yet efficient U-Net-based architecture that directly targets the accuracy–efficiency trade-off in pavement crack segmentation. The proposed model links lightweight design with enhanced feature representation through three complementary modules. First, the feature extraction module (FEM) strengthens shallow spatial feature extraction from raw images and reduces information loss in early feature mapping. Second, the multibranch feature extraction module (MBM) replaces standard convolutional blocks with grouped multibranch convolutions and shared attention, enabling efficient multiscale contextual feature extraction without parameter inflation. Third, the feature aggregation module (FAM) adaptively refines the decoder output to improve final crack prediction. Evaluations on four diverse datasets [18–21] demonstrate that LYEU-Net achieves a favorable balance between segmentation performance and computational efficiency with only 0.134 M parameters and 0.347 GFLOPs. The FEM is designed to extract critical information from raw images, thereby enabling the encoder and decoder to obtain more accurate latent space representations and semantic features. The MBM, which replaces standard convolutional layers, mitigates parameter inflation that is caused by the stacking of numerous convolutional layers. It distributes the input to multiple grouped convolution branches for feature extraction under varying receptive fields, followed by feature merging through a shared-parameter channel-attention mechanism layer. Finally, pointwise convolution and skip connections are used to enrich feature information and improve boundary segmentation capabilities. The FAM, which serves as the output layer, strengthens the representation of the output features by the model. It uses a Hadamard product attention (HPA) mechanism [22] with linear complexity and applies learnable weights to perform Hadamard product operations on the output features for the final predictions. The main contributions of this study are as follows:

1. We propose a lightweight yet effective crack segmentation model that is based on the U-Net architecture and achieves a favorable balance between segmentation performance and computational efficiency. The model contains only 0.134 M parameters and requires 0.347 GFLOPs.
2. A feature extraction module (FEM) is introduced to extract critical information from raw images, thereby minimizing information loss during the mapping of inputs to latent space representations.
3. A multibranch feature extraction module (MBM) is designed to reduce the number of model parameters while efficiently capturing and fusing multiscale features through grouped convolutions and shared attention mechanisms.
4. A feature aggregation module (FAM) is proposed as the output layer to improve the representation of the output features using a Hadamard product attention (HPA) mechanism.
5. The experimental results on four datasets with different environmental disturbances and crack morphologies demonstrate that the proposed method has the fewest parameters and achieves the best mIoU among the compared methods, while showing a precision–recall trade-off on some datasets.

The remainder of this paper is organized as follows. The Related Work section reviews lightweight networks and crack segmentation models. The Proposed Method section introduces the proposed LYEU-Net architecture and describes the FEM, MBM, FAM, and loss function in detail. The Experiment section presents the experimental settings, datasets, evaluation metrics, comparison results, complexity analysis, and ablation study. Finally, the Conclusion section summarizes the findings, discusses limitations, and outlines future research directions.

## Related work

### Lightweight networks

U-Net remains a representative architecture because of its ability to capture both the global context and fine-grained local details. It effectively integrates multiscale information via skip connections, thereby mitigating information loss in deeper layers. However, its original encoder uses standard convolutions, which results in relatively high computational costs. Moreover, the local nature of convolution operations limits the ability of U-Net to model long-range dependencies. Consequently, U-Net and its variants have become popular foundations for end-to-end pixel-level crack segmentation and have often served as bases for integrating lightweight modules or attention mechanisms to increase performance. RASNet [12] introduces a multiscale spatial perception module and a decoding module with attention connections into the U-Net architecture to enrich semantic information. kMaXU [23] is a hybrid encoder and dual-branch decoder architecture that combines a CNN and a Transformer to handle category-imbalanced segmentation tasks. SwinUNet [24] abandons traditional convolution in favor of hierarchical feature learning via shifted window-based Swin Transformers. It uses a patch-expanding layer and symmetric architecture to achieve precise spatial predictions. To reduce the computational cost while preserving contextual information, Yu et al. [25] employed depthwise separable convolutions in the decoder for efficiency. An adjacent layer fusion module was used to enhance multiscale features via skip connections, whereas a dual-channel attention module and a final feature refinement module were used to improve feature quality and segmentation accuracy. Al-Maqtari et al. [5] proposed a fusion of two traditional edge detection methods to create a trainable model through convolutional layers that directly learns edge features without the need to train edge labels separately and with minimal parameters. Zim et al. [26] introduced the ultralightweight subspace attention module, which generates a single attention map per feature subspace using depthwise convolutions followed by a pointwise convolution, thereby reducing computational complexity while preserving performance. Zhang et al. [27] proposed a cross-visual Mamba feature extraction approach that operates without relying on attention mechanisms and increases the accuracy of tiny crack detection by extracting global features through progressive processing and cross-fusion of multilevel feature information.

Recent studies have also explored multichannel feature extraction and attention-based fusion in structural health monitoring. Chen et al. [28] proposed a multichannel CNN with decision-level fusion for multipart cover fault diagnosis, while Chen et al. [29] integrated multihead attention into a Siamese CNN-BiMGRU framework for fault detection under small-sample conditions. These studies further indicate the effectiveness of feature fusion and attention mechanisms for infrastructure inspection tasks.

### Crack segmentation models

Most traditional image segmentation methods are based on thresholding and edge detection techniques [30], which rely on handcrafted rules. However, these approaches struggle to handle crack images with complex structures and fine details, which makes them unsuitable for current crack segmentation scenarios. With the advancement of deep learning, methods that are based on deep neural networks have gradually become mainstream. Among these architectures, convolutional neural network (CNN)-based architectures are the most classic. In OUR-Net [31], the octave convolution residual block in the encoder replaces conventional convolutional layers, and octave max unpooling is used in the decoder as the upsampling operation. This design enables the network to more effectively decode multispatial frequency features, thereby improving its ability to process multiscale information and reduce spatial redundancy. Dong et al. [32] presented a feature coupling encoder in which a CNN branch extracts local features from similar regions in the crack image and a Transformer branch captures global information. These features are coupled to improve global perception and local detail extraction. Zhang et al. [7] designed a network on the basis of a differential convolution encoder and hybrid CNN-Mamba multiscale attention. Enhanced convolution modules and difference convolution operators were introduced to extract the edge information of cracks. Song et al. [33] proposed a two-stage framework for enhancing fine crack features. In the pretraining stage, contrastive learning is applied to unlabeled data, which enables the model to distinguish cracks from the background in a high-dimensional space. During fine-tuning, the model leverages limited labeled data to achieve strong performance without the need for additional annotations. Wu et al. [34] proposed a semantic segmentation model that fuses multipath features to achieve high-precision image segmentation. Ni et al. [35] leveraged large kernel convolution and multiscale feature extraction to capture more detailed pavement crack information during the training phase. To address the issue of imbalanced data distribution, Fan et al. [36] introduced a joint learning loss that combines the Dice and cross-entropy losses with dynamic weighting, where the loss weights are updated on the basis of the learning stages of the model. This adaptive approach improves the recall for underrepresented classes while reducing the risk of overfitting to dominant classes.

However, existing approaches face limitations in terms of balancing accuracy and efficiency. High-performance models achieve excellent results but are unsuitable for mobile deployment. Lightweight models reduce costs but sacrifice performance. Unlike these approaches, LYEU-Net achieves superior performance with only 0.134M parameters through architectural innovations in multiscale feature extraction. The key difference lies in our MBM module, in which grouped convolutions with shared attention mechanisms are used to enable efficient multiscale feature capture without parameter inflation. This distinguishes our work from that of peers by simultaneously optimizing both accuracy and efficiency rather than trading one for the other. Therefore, designing a network that is both lightweight and capable of accurately identifying cracks is essential.

To provide a clearer comparison of existing crack segmentation methods, Table 1 summarizes representative studies in terms of the datasets used, main advantages, and limitations.

**Table 1:** Summary of representative crack segmentation methods.

Method	Datasets	Advantages	Limitations
HED [4]	Crack500	Multiscale edge supervision	Thick predictions and high cost
DeepCrack [37]	Crack500	Deep supervision for crack detection	Relatively large parameter count
LightCrackNet [17]	Crack500	Lightweight architecture	Limited evaluation on diverse datasets
MobileNetv3 [6]	Four datasets used in this study	Efficient lightweight backbone	Lower accuracy on sparse cracks
LMM [5]	Four datasets used in this study	Lightweight and strong recall	Limited precision under complex backgrounds
Qu et al. [3]	Crack500	Multiscale feature fusion	High parameter count
LYEU-Net	Four datasets used in this study	Strong mIoU with 0.134M parameters	Slight recall or F1 decrease on some datasets

## Proposed method

In this section, we detail LYEU-Net, a framework that is derived from the U-Net architecture [8]. The architecture comprises a feature extraction module (FEM), encoder, decoder, and feature aggregation module (FAM) and is designed to achieve an optimal trade-off between model complexity and performance.

### Model architecture

**Fig 2:**
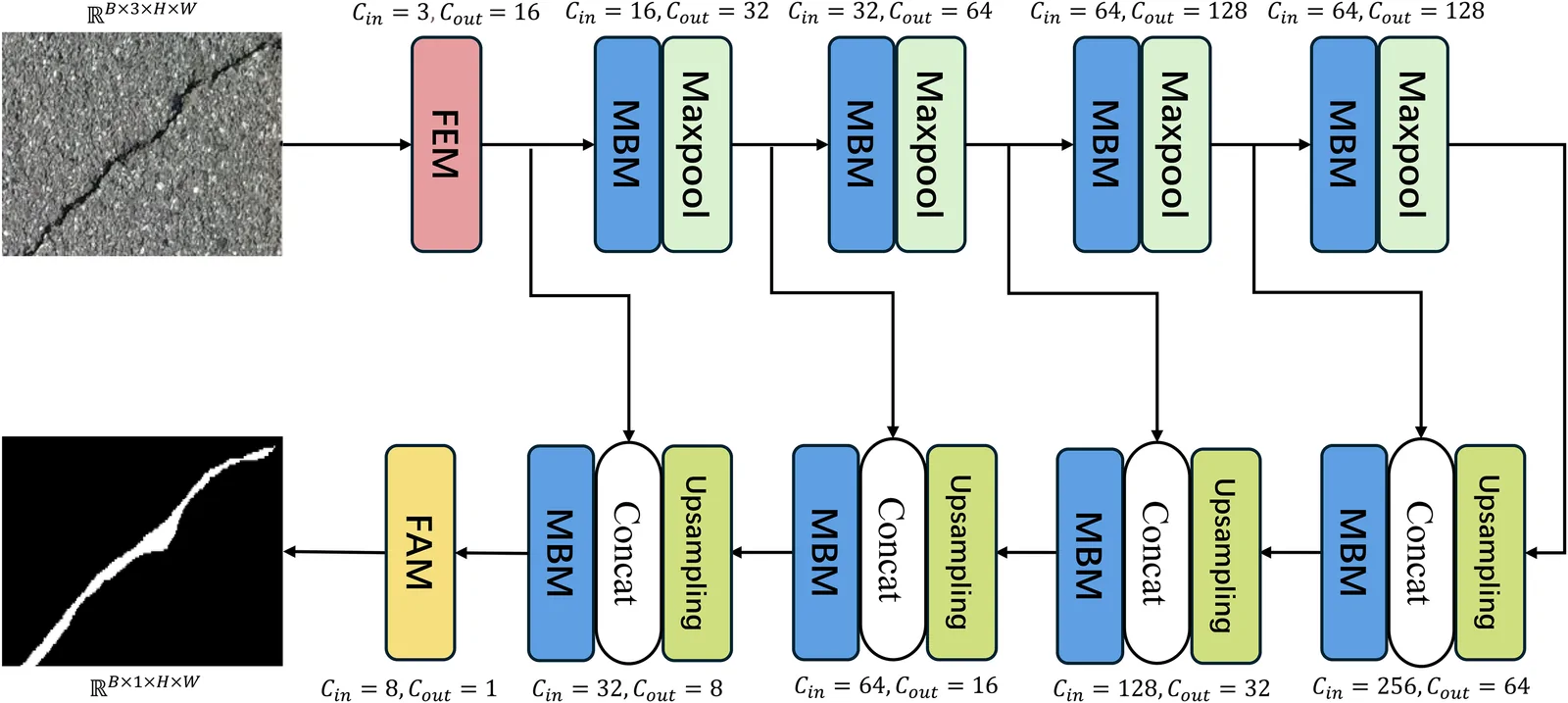
Proposed LYEU-Net architecture.

As illustrated in Fig 2, LYEU-Net comprises an encoder with four downsampling blocks and a decoder with four upsampling blocks. The FEM, which is positioned before the encoder, extracts enriched spatial and semantic representations from raw images while filtering noise that may degrade model performance. Crucially, we replace the standard convolutional operations in the encoder and decoder with MBMs, which leverage grouped convolutions with varying dilation rates to capture multiscale features. Each MBM integrates a shared-parameter channel-attention layer, a pointwise convolution layer, and skip connections to minimize model complexity while increasing boundary segmentation accuracy for intricate cracks. The decoder uses bilinear interpolation to resize high-level features, which are then concatenated with the corresponding encoder features along the channel dimension. The concatenated feature map is directly fed into the subsequent MBM, whose input channels are set to match the concatenated dimension. Channel adjustment and feature fusion are performed by the pointwise convolutions inside the MBM; therefore, no additional pointwise convolution is applied immediately after concatenation. Finally, a feature aggregation module (FAM) is appended after the decoder to refine the final output through adaptive feature weighting, which enables the model to dynamically prioritize the salient regions in the feature maps.

### Feature extraction module

**Fig 3:**
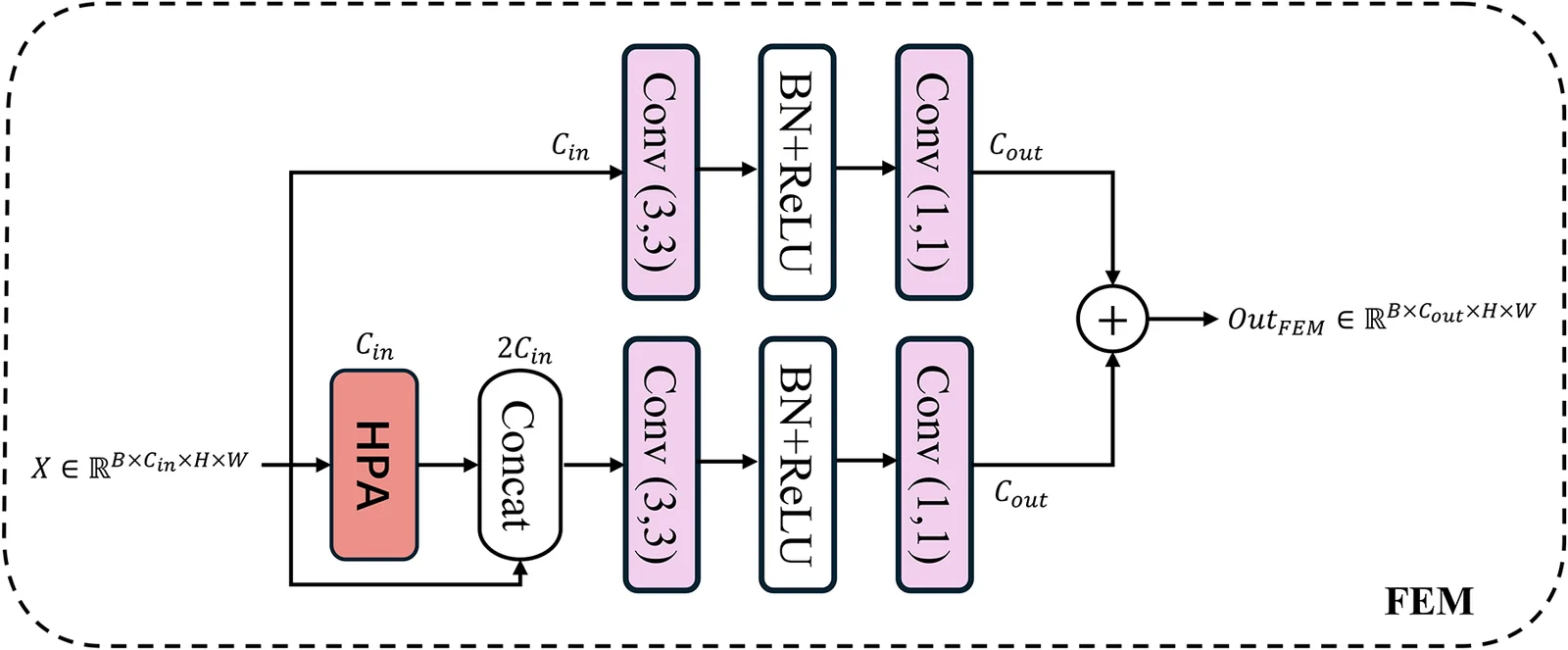
Feature extraction module.

The internal structure of the FEM, which operates directly on raw input images, is depicted in Fig. 3. The FEM consists of two parallel branches. In the upper branch, the input feature map *X* is directly fed into a depthwise separable convolution (DWConv) to generate $Out_{FEM}^{1}$. In the lower branch, *X* is first processed by the Hadamard product attention (HPA) mechanism with linear complexity, and the resulting attention-enhanced feature is concatenated with the original input *X*. The concatenated feature is then fed into another DWConv to generate $Out_{FEM}^{2}$. Finally, $Out_{FEM}^{1}$ and $Out_{FEM}^{2}$ are additively fused to obtain the final FEM output $Out_{FEM}$. In Fig. 3, each DWConv is implemented by a 3×3 convolution followed by batch normalization, ReLU activation, and a 1×1 convolution. Notably, the HPA here differs from the method proposed in [22]. Specifically, we initialize a learnable tensor $S\in {\mathbb{R}}^{1\times C_{in}\times H\times W}$, where $C_{in}$ denotes the number of channels of *X*, and *H* and *W* represent the height and width of the feature map, respectively. The tensor *S* is refined via convolution, and the Hadamard product operation is applied between *X* and the refined tensor to produce the attention-enhanced feature. This process is formulated as follows:

1. Initialization of the learnable tensor:
  a. Input feature map: $X\in {\mathbb{R}}^{B\times C_{in}\times H\times W}$, where *B* represents the batch size and $C_{in}$ represents the number of input channels.
  b. Learnable spatial weight tensor: $S\in {\mathbb{R}}^{1\times C_{in}\times H\times W}$.
2. Computation of $Out_{FEM}^{1}$ in the direct convolution branch:
  $$\begin{array}{c} Out_{FEM}^{1}=W_{1\times 1}^{\left(1\right)}\left(\sigma \left(BN\left(W_{3\times 3}^{\left(1\right)}\left(X\right)\right)\right)\right), \end{array}$$ (1)
  where $W_{3\times 3}^{\left(1\right)}$ represents a 3×3 convolution, BN denotes batch normalization, σ represents the ReLU activation function, and $W_{1\times 1}^{\left(1\right)}$ represents a 1×1 convolution with $C_{out}$ output channels.
3. Computation of $Out_{FEM}^{2}$ in the HPA-concatenation branch:
  a. Transform the learnable tensor:
    $$\begin{array}{c} S^{\prime }=W_{1\times 1}^{\left(2\right)}\left(\sigma \left(BN\left(W_{3\times 3}^{\left(2\right)}(S)\right)\right)\right), \end{array}$$ (2)
    where $W_{3\times 3}^{\left(2\right)}$ represents a 3×3 convolution, and $W_{1\times 1}^{\left(2\right)}$ represents a 1×1 convolution.
  b. Hadamard product:
    $$\begin{array}{c} Out_{HPA}=X⊙S^{\prime }, \end{array}$$ (3)
    where ⊙ denotes elementwise multiplication.
  c. Channel concatenation:
    $$\begin{array}{c} X^{\prime }=\mathrm{Concat}\left(Out_{HPA},X\right)\in {\mathbb{R}}^{B\times 2C_{in}\times H\times W}. \end{array}$$ (4)
  d. Generate $Out_{FEM}^{2}$:
    $$\begin{array}{c} Out_{FEM}^{2}=W_{1\times 1}^{\left(3\right)}\left(\sigma \left(BN\left(W_{3\times 3}^{\left(3\right)}\left(X^{\prime }\right)\right)\right)\right), \end{array}$$ (5)
    where $W_{3\times 3}^{\left(3\right)}$ represents a 3×3 convolution and $W_{1\times 1}^{\left(3\right)}$ represents a 1×1 convolution with $C_{out}$ output channels.
4. Final output:
  $$\begin{array}{c} Out_{FEM}=Out_{FEM}^{1}+Out_{FEM}^{2}\in {\mathbb{R}}^{B\times C_{out}\times H\times W}, \end{array}$$ (6)
  where $C_{out}$ represents the number of output channels.

### Multibranch feature extraction module

**Fig 4:**
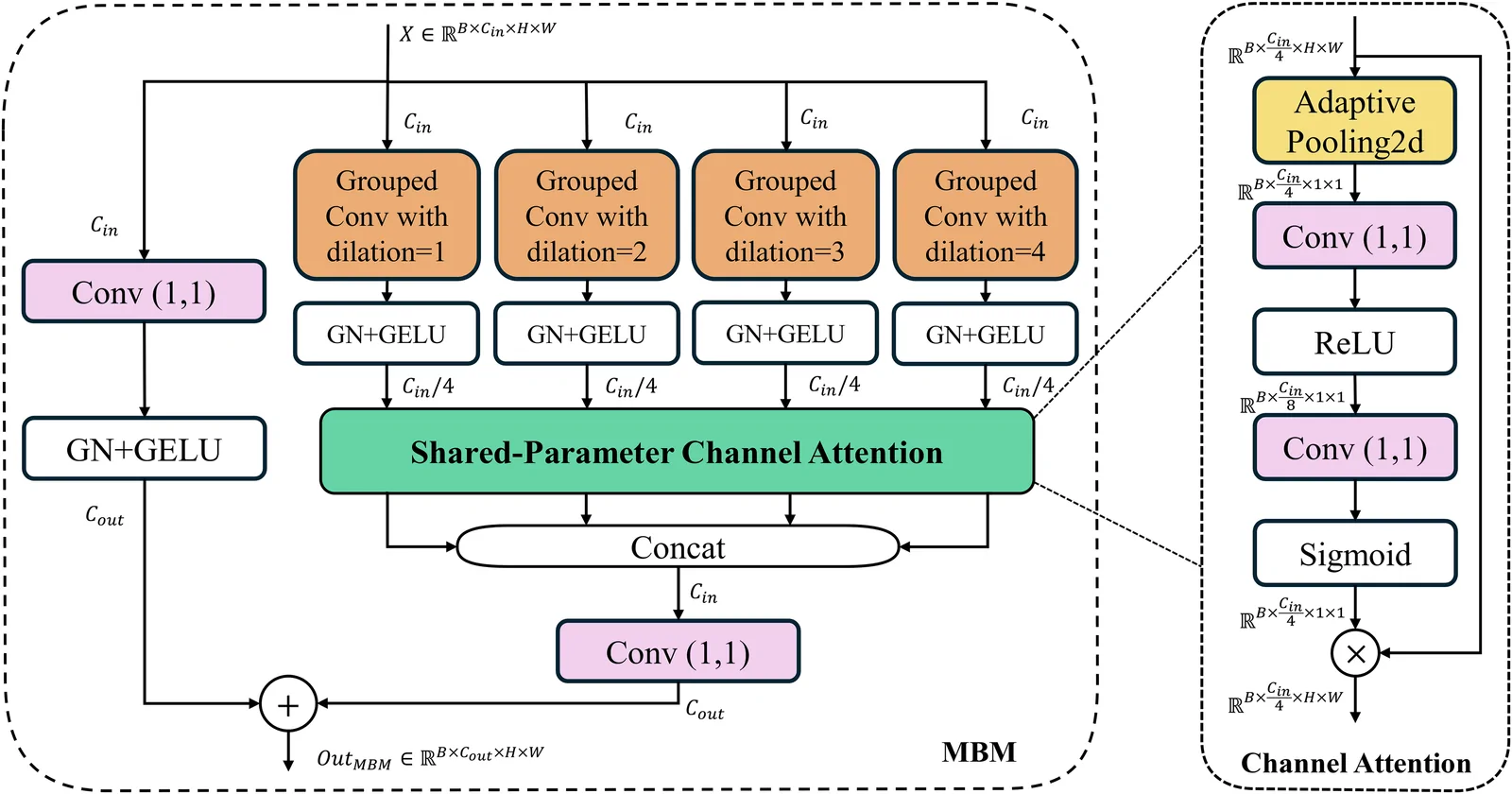
Multibranch feature extraction module.

Crack image segmentation is a dense prediction task that is widely recognized to critically depend on the extraction of multiscale information [38]. Traditional convolutional layers capture spatial, semantic, and contextual information through stacking, but this approach introduces a substantial number of parameters and reduces model robustness. To address this, we propose MBMs to replace conventional convolutional layers, which enable improved multiscale feature extraction while reducing the number of model parameters, as shown in Fig 4. First, the MBM processes the input *X* using four grouped convolution branches with varying dilation sizes to capture multiscale features. Subsequently, a shared-parameter channel attention mechanism is applied to increase the focus on feature channels across different scales. The shared parameter mechanism in the channel attention layer reduces the model complexity. The MBM then concatenates the four multiscale feature maps along the channel dimension and correlates the multiscale information through pointwise convolution, with the output dimensions adjusted to obtain ${Out}_{MBM}^{1}\in {\mathbb{R}}^{B\times C_{out}\times H\times W}$. Additionally, the MBM uses a skip connection with pointwise convolution to mitigate feature loss, which results in ${Out}_{MBM}^{2}\in {\mathbb{R}}^{B\times C_{out}\times H\times W}$. Finally, the outputs ${Out}_{MBM}^{1}$ and ${Out}_{MBM}^{2}$ are fused via elementwise addition to generate the final output ${Out}_{MBM}$. This process is summarized as follows:

1. Input feature map: $X\in {\mathbb{R}}^{B\times C_{in}\times H\times W}$, where *B* represents the batch size and $C_{in}$ represents the number of input channels.
2. Multibranch Feature Extraction: For each branch *i* = 1,2,3,4:
  $$\begin{array}{c} F_{i}=GELU\left({GN}_{4}\left({GroupConv}_{d=i}(X)\right)\right), \end{array}$$ (7)
  where ${GroupConv}_{d=i}$ denotes a grouped convolution with $groups=C_{in}/4$ and dilation rate *d*={1,2,3,4}, *GN*_4_ represents group normalization with $num_{g}roups=C_{in}/4$, and *GELU* represents the Gaussian error linear unit activation function.
3. Shared-Parameter Channel Attention: For each branch feature $F_{i}\in {\mathbb{R}}^{B\times \left(C_{out}/4\right)\times H\times W}$:
  $$\begin{array}{c} F_{i}^{\prime }=\sigma \left(W_{2}\cdot ReLU\left(W_{1}\cdot GAP\left(F_{i}\right)\right)\right)⊙F_{i}, \end{array}$$ (8)
  where *GAP* represents global average pooling, $W_{1}\in {\mathbb{R}}^{\left(C_{in}/8\right)\times \left(C_{in}/4\right)}$ and $W_{2}\in {\mathbb{R}}^{\left(C_{in}/4\right)\times \left(C_{in}/8\right)}$ represent learnable 1×1 convolutional weights, σ represents the sigmoid function and ⊙ denotes elementwise multiplication.
4. Feature Fusion and Output:
  a. Multiscale Feature Fusion:
    $$\begin{array}{c} F_{fused\;}=Concat\left(F_{1}^{\prime },F_{2}^{\prime },F_{3}^{\prime },F_{4}^{\prime }\right)\in {\mathbb{R}}^{B\times C_{in}\times H\times W}, \end{array}$$ (9)
  b. Pointwise Convolution for Dimensionality Adjustment:
    $$\begin{array}{c} {Out}_{MBM}^{1}=W_{out\;}\cdot F_{fused\;}, \end{array}$$ (10)
    where $W_{out\;}\in {\mathbb{R}}^{C_{out}\times C_{in}\times 1\times 1}$.
  c. Skip Connection and Final Output:
    $$\begin{array}{c} {Out}_{MBM}^{2}=W_{skip}\cdot X, \end{array}$$ (11)
    $$\begin{array}{c} {Out}_{MBM}={Out}_{MBM}^{1}+{Out}_{MBM}^{2}\in {\mathbb{R}}^{B\times C_{out}\times H\times W}, \end{array}$$ (12)
    where $W_{skip}\in {\mathbb{R}}^{C_{out}\times C_{in}\times 1\times 1}$.

### Feature aggregation module

**Fig 5:**
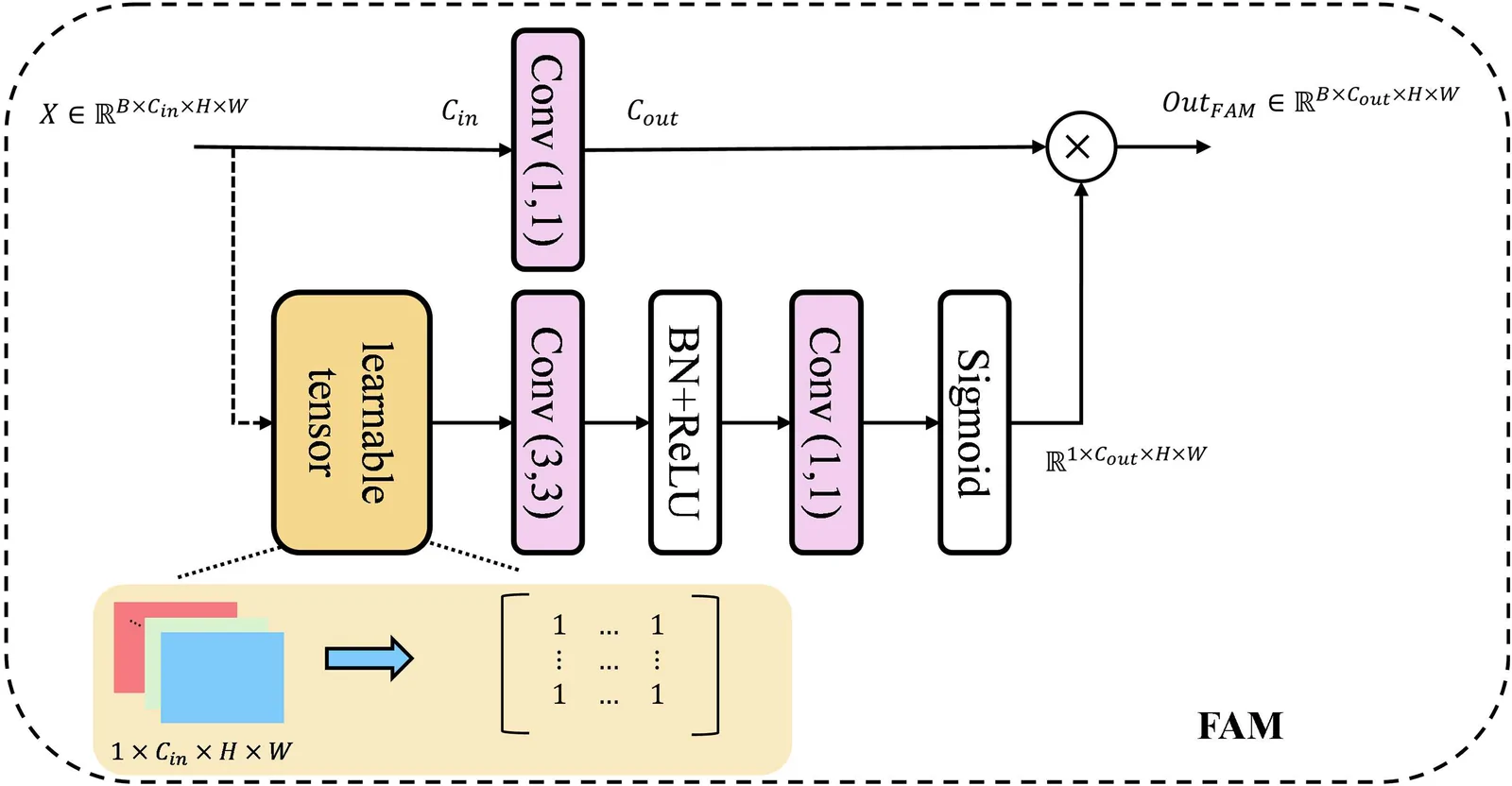
Feature aggregation module.

The feature aggregation module (FAM) processes the output of the decoder and its internal structure is depicted in Fig 5. First, the FAM initializes a learnable tensor $S\in {\mathbb{R}}^{1\times C_{in}\times H\times W}$, where $C_{in}$ represents the number of channels of the input *X* and *H* and *W* represent the height and width, respectively, of the feature maps. Subsequently, *S* undergoes depthwise separable convolution (DWConv) followed by sigmoid activation to produce $S^{\prime }$. Next, the FAM applies a pointwise convolution (PConv) to the input *X*, which generates the intermediate output ${Out}_{P}$. Finally, the Hadamard product operation is performed between ${Out}_{P}$ and $S^{\prime }$ to obtain the final output ${Out}_{FAM}\in {\mathbb{R}}^{B\times C_{out}\times H\times W}$. Because the FAM operates on the final output layer of the model, $C_{out}$ is set to 1 for binary segmentation. This process is formulated as follows:

1. Initialization of the Learnable Tensor:
  a. Input feature map: $X\in {\mathbb{R}}^{B\times C_{in}\times H\times W}$, where *B* represents the batch size and $C_{in}$ represents the number of input channels.
  b. Learnable spatial weight tensor:
    $$\begin{array}{c} S\in {\mathbb{R}}^{1\times C_{in}\times H\times W}. \end{array}$$ (13)
2. Spatial Weight Transformation:
  $$\begin{array}{c} S^{\prime }=\sigma_{sigmoid}\left(W_{1\times 1}^{\left(1\right)}\left(\sigma_{ReLU}\left(BN\left(W_{3\times 3}^{\left(1\right)}(S)\right)\right)\right)\right), \end{array}$$ (14)
  where $W_{3\times 3}^{\left(1\right)}$ represents a 3×3 convolution with $C_{in}/2$ output channels, *BN* denotes BatchNorm, $\sigma_{ReLU}$ represents the ReLU activation function, $W_{1\times 1}^{\left(1\right)}$ represents a 1×1 convolution with $C_{out}$ output channels, and $\sigma_{sigmoid}$ represents the sigmoid activation function.
3. Input Feature Transformation:
  $$\begin{array}{c} {Out}_{P}=W_{1\times 1}^{\left(2\right)}(X), \end{array}$$ (15)
  where $W_{1\times 1}^{\left(2\right)}$ represents a 1×1 convolution with $C_{out}$ output channels.
4. Final Output
  $$\begin{array}{c} {Out}_{FAM}={Out}_{P}⊙S^{\prime }\in {\mathbb{R}}^{B\times C_{out}\times H\times W}, \end{array}$$ (16)
  where ⊙ denotes elementwise multiplication.

### Loss function

The design of loss functions is pivotal for model training, and careful consideration of class imbalance, pixelwise prediction accuracy, and model robustness in crack detection scenarios is needed. Given the complementary nature of different loss functions, we use a joint optimization framework that combines binary cross-entropy (BCE) loss, Dice loss, and mIoU loss during training. BCE loss addresses subtle texture distinctions between cracks and backgrounds, Dice loss mitigates fragmentation in crack regions, and mIoU loss optimizes the overlap between predictions and ground-truth annotations. By integrating these losses, the framework significantly improves the recall and precision in complex road scenarios while preserving boundary delineation. This multiobjective approach reduces the reliance on any single loss function and ensures balanced optimization. The composite loss is defined as follows:


$$\begin{array}{c} {ℒ}_{BCE}=\frac{1}{B}\sum_{b=1}^{B}\left[-\frac{1}{N}\sum_{i=1}^{N}\left(t_{b,i}\cdot \log\left(\sigma \left(p_{b,i}\right)\right)+\left(1-t_{b,i}\right)\cdot \log\left(1-\sigma \left(p_{b,i}\right)\right)\right)\right], \end{array}$$ (17)

where *B* represents the batch size, *N* denotes the total number of pixels per sample (N=H×W), $p_{b,i}$ represents the predicted value of the i-th pixel in the b-th sample, $t_{b,i}$ represents the target value (0 or 1) of the i-th pixel in the b-th sample, and σ represents the sigmoid function.


$$\begin{array}{c} {ℒ}_{Dice\;}=\frac{1}{B}\sum_{b=1}^{B}\left[1-\frac{2\sum_{i=1}^{N}\sigma \left(p_{b,i}\right)\cdot t_{b,i}+\epsilon }{\sum_{i=1}^{N}\sigma \left(p_{b,i}\right)+\sum_{i=1}^{N}t_{b,i}+\epsilon }\right], \end{array}$$ (18)

where $\epsilon =10^{-8}$ represents a smoothing term to prevent division by zero.


$$\begin{array}{c} {ℒ}_{mIoU}=\frac{1}{B}\sum_{b=1}^{B}\left[1-\frac{\sum_{i=1}^{N}\sigma \left(p_{b,i}\right)\cdot t_{b,i}+\epsilon }{\sum_{i=1}^{N}\sigma \left(p_{b,i}\right)+\sum_{i=1}^{N}t_{b,i}-\sum_{i=1}^{N}\sigma \left(p_{b,i}\right)\cdot t_{b,i}+\epsilon }\right]. \end{array}$$ (19)

Finally, the total loss of the model is formulated as the sum of all the individual losses, as shown in Equation (20). This synergistic integration ensures that diverse gradient directions from distinct loss terms complement each other, thereby preventing the model from converging to suboptimal local minima and improving its generalization ability.


$$\begin{array}{c} {ℒ}_{total\;}={ℒ}_{BCE}+{ℒ}_{Dice\;}+{ℒ}_{mIoU}. \end{array}$$ (20)

### Experiment

In this section, the detailed configurations of the model, datasets, baseline comparisons, evaluation metrics, experimental results, ablation study, and complexity analysis are comprehensively discussed. All the information that pertains to the datasets and comparative model results, except for the results of the models reported in references [17] and [3], is derived from reimplementations that are based on publicly accessible code. Owing to the unavailability of code for the models in references [17] and [3], their results are reported on the basis of findings from their original publications.

### Model configuration

We implemented the model using the PyTorch deep learning framework. The feature extraction module (FEM) operates with input and output channel dimensions of 3 and 16, respectively, whereas the feature aggregation module (FAM) operates with input and output dimensions of 8 and 1, respectively. The architecture comprises an encoder with four downsampling blocks and a decoder with four upsampling blocks. The input and output channel dimensions of the encoder are [16,32, 64, 128] and [32, 64, 128, 256], respectively, whereas the input and output dimensions of the decoder are [256, 128, 64, 32] and [64, 8,16,32], respectively. Additional implementation details are available in our publicly shared code.

For optimization, we used the AdamW optimizer. The hyperparameter settings that were used during training are summarized in Table 2. The learning rate was reduced by a factor of 0.1 if no loss decrease was observed for 10 consecutive epochs. The key optimizer parameters included a weight decay of 0.01, first-moment decay (beta1) of 0.9, second-moment decay (beta2) of 0.999, and epsilon of 1e-8. Model checkpoints were saved whenever a new peak mIoU score was achieved.

**Table 2:** Model hyperparameter settings.

Learning rate	Epochs	Batch size	Optimizer
0.01	120	8	AdamW

All the experiments were conducted on a desktop computer running Windows 10 with Python 3.12. The hardware configuration included a 12th Gen Intel Core i9-12900K 3.19 GHz CPU (2 CPUs) and a 24 GB NVIDIA GeForce RTX 3090 GPU.

### Dataset

To ensure fair comparative evaluations, we preprocessed the original datasets using the methodology described in [5], which primarily involves cropping the raw images into smaller crack images. During data loading, all cropped images were uniformly resized to 256 × 256 pixels before being fed into the network. Therefore, the size of 256 × 256 refers to the resized network input size rather than the original cropping patch size. The Train/Valid/Test split reported in Table 3 refers to the number of cropped images after preprocessing rather than the number of original raw images. The Crack500 dataset [18] consists of images of pavement cracks that were captured using smartphones and feature complex backgrounds and diverse crack types. The GAPs384 dataset [19] contains a large volume of manually annotated asphalt crack images from German roadways. The AigleRN-TRIMM dataset [20] includes both real and simulated crack images, although our experiments exclusively used real images. The ShadowCrack dataset [21] comprises pavement crack images that were captured under varying illumination conditions, with shadowed regions particularly emphasized. These datasets collectively validate the generalization ability of the model for crack detection under diverse scenarios, pavement backgrounds, crack morphologies, and lighting conditions. Details on the partitioned training, validation, and test sets, along with the crack-to-pixel ratios, are provided in Table 3.

**Table 3:** Dataset details.

Datasets	Size			Crack-to-pixel Ratios
	Train	Valid	Test	
Crack500	5270	899	4128	6.03%
GAPs384	942	157	611	1.21%
AigleRN-TRIMM	152	39	78	1.50%
ShadowCrack	406	140	125	2.00%

### Baselines

In selecting models for comparison, we chose seven lightweight models with fewer than 15M trainable parameters and one high-parameter model, Qu et al. [3], which contains 58.72M trainable parameters:

1. Qu et al. [3]: A multiscale convolutional feature fusion module for crack detection.
2. HED [4]: An edge detection algorithm that addresses holistic image training and multiscale feature learning.
3. DeepCrack [37]: An end-to-end crack detection model that combines an extended fully convolutional network (FCN) and a deeply supervised network (DSN).
4. EfficientNet [39]: A novel model scaling method and a family of architectures. We selected EfficientNet-b0 with 6.97M trainable parameters for comparison.
5. LightCrackNet [17]: A lightweight crack detection model with merely 1.329M trainable parameters.
6. ShuffleNetv2 [40]: A speed-accuracy balanced architecture that was designed by optimizing memory access costs and platform characteristics.
7. MobileNetv3 [6]: A semantic segmentation model that was developed through the codesign of automated search algorithms and network architectures.
8. LMM [5]: A modular lightweight crack segmentation model with only 0.87M trainable parameters.

### Evaluation metrics

In the empirical analysis phase, we conducted model validation experiments using the aforementioned datasets. For each training batch, the system computed four evaluation metrics, namely, the recall, precision, F score, and mIoU, to quantify the model performance. After each iteration, these metrics were recalculated on the validation set to determine checkpoint savings. The F score, which is the harmonic mean of precision and recall, effectively reflects the balanced performance of the model in classifying positive and negative samples. The mean intersection over union (mIoU) measures the segmentation accuracy by comparing the overlap between the predictions and ground-truth masks. Both metrics are derived from statistical counts of true positives (TP), true negatives (TN), false positives (FP), and false negatives (FN) by following established mathematical formulations. In all subsequent experiments, the classification threshold was set to 0.5. The metrics are defined as follows:


$$\begin{array}{c} Precision=\frac{TP}{TP+FP}, \end{array}$$ (21)

$$\begin{array}{c} Recall=\frac{TP}{TP+FN}, \end{array}$$ (22)

$$\begin{array}{c} F-score=2*\frac{Precision*Recall}{Precision+Recall}, \end{array}$$ (23)

$$\begin{array}{c} IoU=\frac{TP}{TP+FN+FP}. \end{array}$$ (24)

### Experimental results

**Fig 6:**
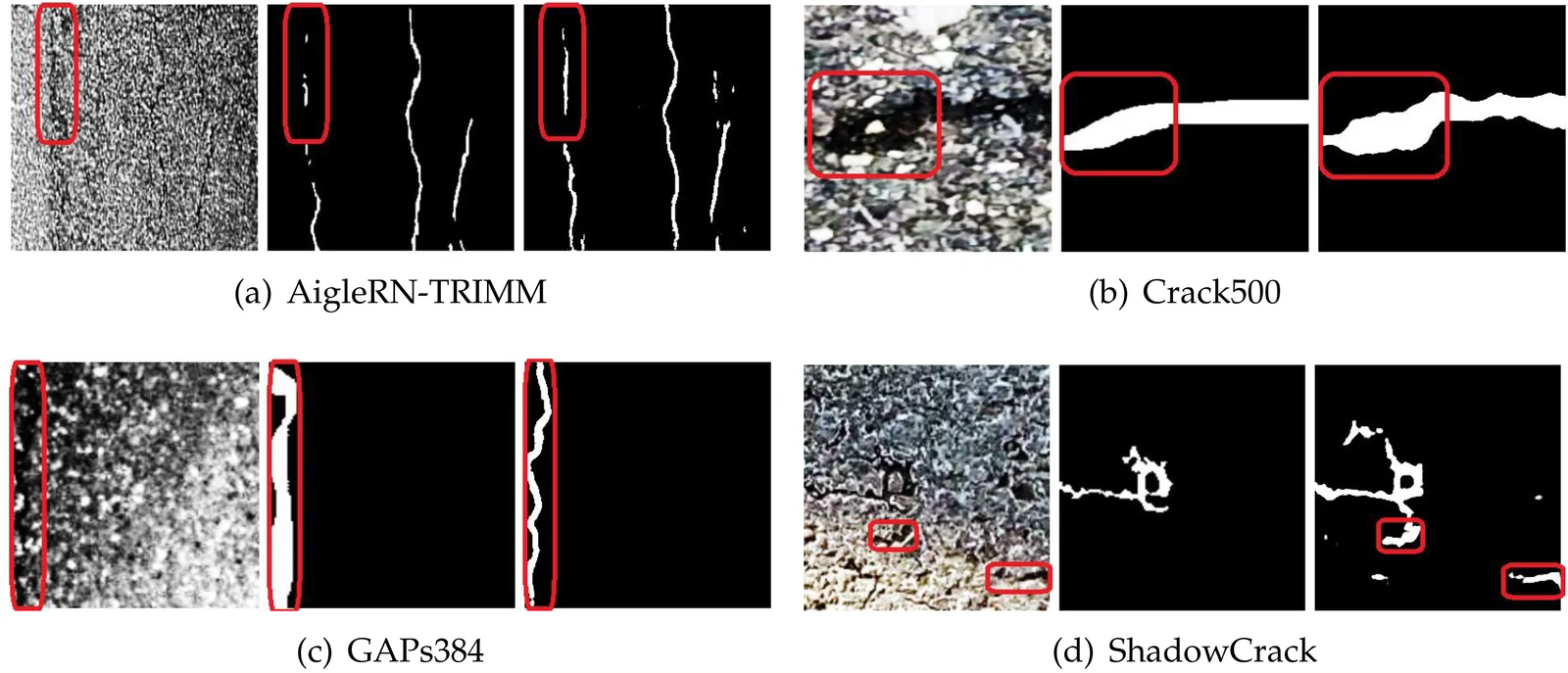
LYEU-Net segmentation results on various datasets.

For each dataset, the model was trained on its respective training set, with the best-performing checkpoint selected according to the validation set and ultimately evaluated on the independent test set. The experiments followed the fixed train/validation/test split protocol used in previous crack segmentation studies to ensure fair comparison with existing methods. Although K-fold cross-validation was not conducted in this study, the use of four datasets with different crack morphologies, pavement backgrounds, illumination conditions, and crack-to-pixel ratios provides a broad evaluation of the generalization ability of the proposed model. First, we analyzed the output of the model qualitatively. The representative segmentation results across the four datasets are shown in Fig 6. Each subfigure displays, from left to right, the original image, ground truth, and segmentation results.

Notably, manual labeling in the original datasets inevitably introduces annotation errors. Our model occasionally outperformed the ground-truth annotations, as evidenced by the following: unlabeled continuous cracks in AigleRN-TRIMM, partially labeled wide cracks in Crack500, mislabeled narrow cracks in GAPs384, and unannotated crack clusters in ShadowCrack. These cases, which were successfully detected by LYEU-Net, highlight its ability to address crack omission and complex crack morphologies and demonstrate its increased sensitivity to crack presence even under imperfect supervision.

We conducted performance comparisons between LYEU-Net and the other models across all the datasets. Bold numbers in the tables denote the best results for each metric, and “mean” represents the average of recall, precision, F score, and mIoU. As shown in Table 4, on Crack500, LYEU-Net improved precision and mIoU by 5.7% and 0.5%, respectively, compared with LMM. However, its recall and F1 score were lower than those of LMM, decreasing from 80.86% to 77.83% and from 74.89% to 73.74%, respectively. This result indicates that LYEU-Net produces more conservative but more precise crack predictions on Crack500. Compared with DeepCrack (14.72M parameters), LYEU-Net improved the F score by 18.55% and the mIoU by 12.68% while using only 0.91% of its parameters. On ShadowCrack, LYEU-Net achieved the best values for all reported metrics, with relative gains of 19.0% in recall, 29.8% in precision, 18.9% in F1 score, and 8.0% in mIoU compared with LMM. These results indicate that LYEU-Net achieves a favorable balance between segmentation performance and computational efficiency, with a model size of under 1 MB enabling practical mobile deployment.

**Table 4:** Results of all the models on the Crack500 and ShadowCrack test sets.

Model	Crack500					ShadowCrack					Params(M)
	R	P	F1	mIoU	mean	R	P	F1	mIoU	mean	
Qu et al. [3]	69.8	65.4	67.5	73.5	69.05	–	–	–	–	–	58.72
HED [4]	71.21	70.78	70.99	72.12	71.28	39.56	43.09	41.25	60.12	46.01	14.71
DeepCrack [37]	44.2	73.46	55.19	62.54	58.85	6.2	62.95	11.22	50.84	32.79	14.72
EfficientNet [39]	77.03	57.23	65.67	66.07	66.5	33.65	27.1	30.02	55.35	36.53	6.97
LightCrackNet [17]	72.1	75.3	73.7	74.5	73.9	–	–	–	–	–	1.329
ShuffleNetv2 [40]	73.64	67.7	70.54	71.3	70.8	37.37	33	35.07	57.4	40.72	1.38
MobileNetv3 [6]	76.28	62.49	68.7	69.47	69.24	28.01	24.18	26	53.85	33	1.06
LMM [5]	**80.86**	69.74	**74.89**	74.81	75.08	52.88	43.75	47.88	63.02	51.88	0.87
Ours	77.83	**73.71**	73.74	**75.22**	**75.13**	**62.92**	**56.8**	**56.95**	**68.06**	**61.17**	**0.134**

Table 5 presents the results on the GAPs384 and AigleRN-TRIMM datasets. On GAPs384, LYEU-Net outperformed LMM by 28.7% in precision, 7.8% in F score, and 5.0% in mIoU. However, its recall was slightly lower than that of LMM, decreasing from 60.66% to 60.12%. On AigleRN-TRIMM, LYEU-Net achieved improvements of 12.3% in precision, 4.0% in F score, and 2.8% in mIoU compared with LMM, although its recall was slightly lower. Across all four datasets, LYEU-Net achieved the best mIoU among the compared methods and maintained the smallest parameter count. These results show that LYEU-Net does not uniformly outperform all baselines on every metric but provides a strong accuracy–efficiency balance, with a tendency toward higher precision and mIoU at the cost of slightly reduced recall on some datasets.

**Table 5:** Results of all the models on the GAPs384 and AigleRN-TRIMM test sets.

Model	GAPs384					AigleRN-TRIMM					Params(M)
	R	P	F1	mIoU	mean	R	P	F1	mIoU	mean	
Qu et al. [3]	–	–	–	–	–	–	–	–	–	–	58.72
HED [4]	21.09	62.52	31.54	57.81	43.24	10.23	59.43	17.46	53.65	35.19	14.71
DeepCrack [37]	3.9	65.71	7.27	50.42	31.83	2.77	99.7	5.4	50.35	39.56	14.72
EfficientNet [39]	46.73	30.53	36.93	58.63	43.21	36.15	21.76	27.17	55.76	35.21	6.97
LightCrackNet [17]	–	–	–	–	–	–	–	–	–	–	1.329
ShuffleNetv2 [40]	51.22	43.6	47.12	63.3	51.31	55.58	31.99	40.61	61.01	47.3	1.38
MobileNetv3 [6]	50.43	31.09	38.5	59.11	44.78	29.89	19.6	23.64	54.58	31.93	1.06
LMM [5]	**60.66**	46.65	52.74	65.87	56.48	**78.34**	64.74	70.9	76.67	72.66	0.87
Ours	60.12	**60.04**	**56.83**	**69.19**	**61.55**	76.68	**72.68**	**73.75**	**78.81**	**75.48**	**0.134**

We analyzed the FLOPs and parameter counts of all the models, and the detailed results are presented in Table 6. Compared with the other models, our architecture achieved the lowest parameter count, with more than 50% fewer parameters than the LMM. This efficiency stemmed primarily from the replacement of redundant convolutional layers with the highly optimized MBM. Although MobileNetv3 had fewer FLOPs, which was attributed to its fewer convolutional kernels and smaller feature map dimensions (H, W), such optimizations compromise segmentation performance.

**Table 6:** Complexity comparison of all the models.

Models	FLOPs(G)	Params (M)
Qu et al. [3]	73.75	58.72
HED [4]	15.4	14.71
DeepCrack [37]	15.42	14.72
EfficientNet [39]	0.906	6.97
LightCrackNet [17]	8.01	1.329
ShuffleNetv2 [40]	1.105	1.38
MobileNetv3 [6]	**0.132**	1.06
LMM [5]	6.56	0.87
**Ours**	0.347	**0.134**

**Fig 7:**
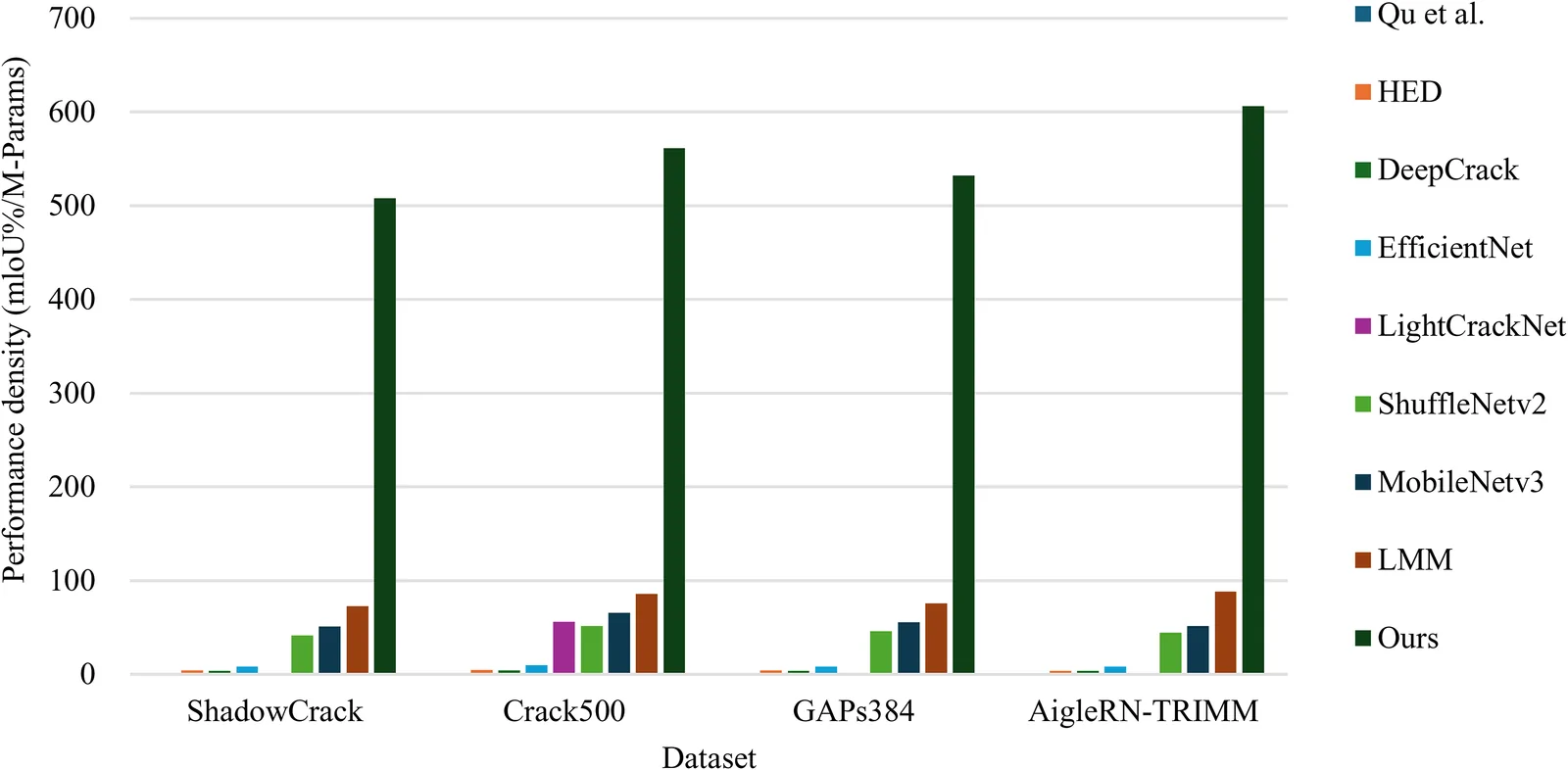
Efficiency metrics of all the models on each dataset.

As illustrated in Fig 7, in which efficiency metrics are compared across models, our architecture demonstrated significantly superior performance density per million parameters, which indicates an optimal balance between parameter utilization and model efficacy.

Notably, the inference speed (FPS) that is reported in this study was evaluated on a standard desktop GPU (RTX 3090) to establish a theoretical baseline for efficiency. While the ultralow FLOPs (0.347G) and parameter count (0.134M) strongly suggest suitability for mobile deployment, direct benchmarking on embedded hardware (e.g., Jetson Nano or FPGA) was not conducted in this work because of hardware limitations. Validating the real-time performance on such edge devices remains a critical objective for our future research.

### Ablation study

Our model uses the proposed feature extraction module (FEM), multibranch feature extraction module (MBM), and feature aggregation module (FAM). To analyze the effect of each module on the overall performance, we replaced these modules with standard convolutional operations while maintaining their input and output dimensions to establish a base model. Specifically, the FEM was substituted with a 3× 3 convolutional layer followed by a 1× 1 convolutional layer, with batch normalization (BN) and ReLU activation inserted between the two layers. The MBM was replaced with two 3× 3 convolutional layers, followed by BN and ReLU activation. The FAM was replaced with a 1× 1 convolutional layer. For instance, “B + MBM” denotes the base model with only the MBM retained and the FEM and FAM replaced with standard convolutions.

The ablation study was conducted on the ShadowCrack dataset because this dataset contains strong shadow interference, varying illumination, low contrast, cluttered backgrounds, and irregular crack patterns, making it one of the most challenging datasets for evaluating the effectiveness of the proposed modules. Therefore, ShadowCrack provides a representative difficult scenario for assessing whether the FEM, MBM, and FAM improve feature extraction, multiscale representation, and output refinement under complex real-world conditions. The purpose of this ablation study is to isolate the contribution of each module under a challenging setting, whereas the complete LYEU-Net is evaluated on all four datasets to verify its overall generalization ability.

As shown in Table 7, the ablation results on the ShadowCrack dataset reveal the following: Compared with the base model, the FEM improved the precision and mIoU, indicating reduced crack misclassification rates. The FAM improved all the metrics and demonstrated improved model stability and reliability. The MBM achieved significant gains across all the metrics while reducing the numbers of FLOPs and parameters by 73.18% and 84.05%, respectively. This highlights its dual role in parameter efficiency and performance optimization, which enables more accurate classification and precise boundary localization under challenging shadow conditions. By integrating all three modules, our model reduced the numbers of FLOPs and parameters from 1.223G and 0.834M to 0.347G and 0.134M, respectively, while achieving a 5.72% average improvement across all the metrics. Although this ablation study was conducted on a single challenging dataset, the complete model was further evaluated on all four datasets to assess its overall generalization ability. More extensive cross-dataset ablation studies will be considered in future work.

**Table 7:** Ablation results of our model on ShadowCrack.

Models	R	P	F1	mIoU	mean	FLOPs(G)	Params (M)
Base (B)	59.25	53.5	52.67	66	57.86	1.223	0.834
B + FEM	57.74	54.75	52.55	66.11	57.79	1.226	0.83
B + FAM	59.31	54.53	52.92	66.24	58.25	1.238	0.834
B + MBM	61.09	**57.07**	56.07	67.67	60.48	**0.328**	**0.133**
Ours	**62.92**	56.75	**56.95**	**68.06**	**61.17**	0.347	0.134

## Conclusion

In this paper, LYEU-Net, which is a lightweight crack segmentation model that successfully resolves the conflict between segmentation accuracy and computational efficiency, is presented. By integrating the feature extraction module (FEM), multibranch feature extraction module (MBM), and feature aggregation module (FAM), the proposed architecture captures rich multiscale features with minimal parameters. This work advances the body of knowledge by demonstrating that efficient feature aggregation can replace massive parameter scaling. Unlike high-performance models that require heavy computational resources or lightweight models that compromise segmentation quality, LYEU-Net achieves a strong balance between accuracy and efficiency with only 0.134M parameters. In particular, it obtains the best mIoU among the compared methods on all four datasets and demonstrates competitive overall segmentation performance. Nevertheless, slight decreases in recall or F1 score occur on some datasets, reflecting a precision–recall trade-off. The research implications are significant in two aspects. Theoretically, this study diverges from the prevailing trend of increasing model depth and provides empirical evidence that structural optimization–specifically grouped convolutions with shared attention–is more effective for texture-dependent tasks. In practice, the sub-1 MB model size enables immediate deployment on edge devices for real-time infrastructure monitoring, thereby effectively bridging the gap between academic algorithms and industrial engineering needs.

Despite these achievements, certain limitations remain. First, the current architecture lacks a dedicated edge-extraction branch. Although the FAM improves the global context, this omission can lead to minor imprecision in delineating the boundaries of large-scale cracks, particularly in low-contrast scenarios in which segmentation boundaries may appear blurred. Second, although the theoretical computational cost (0.347G FLOPs) indicates high efficiency, GPU-based simulations were relied on in this study, and direct inference benchmarking was not performed on specific embedded hardware (e.g., FPGA or Jetson devices). Third, the experiments followed a fixed train/validation/test split protocol rather than K-fold cross-validation. Although the model was evaluated on four datasets with diverse crack morphologies and imaging conditions, more extensive repeated experiments or cross-validation would further strengthen the statistical reliability of the results. Additionally, the adaptability of the model to other structural defects beyond pavement cracks, such as those on bridges or building walls, remains to be verified.

Future research will prioritize addressing these limitations. We aim to integrate a lightweight edge-supervision branch that uses gradient information or contour cues to sharpen boundary localization without significantly increasing the parameter count. Furthermore, we will conduct comprehensive hardware-in-the-loop testing to validate real-world inference speeds on mobile platforms. Subsequent work will also explore the application of the model in joint crack detection and classification tasks, as well as multimodal data fusion. In addition, more extensive cross-dataset ablation studies will be conducted to further examine the generalizability of the proposed modules under different crack morphologies and environmental conditions. Finally, collaborations with transportation departments are planned to conduct field tests under extreme lighting conditions, thereby promoting the transformation of LYEU-Net into a robust engineering solution.

## References

[1] Lin Q, Li W, Zheng X, Fan H, Li Z. DeepCrackAT: An effective crack segmentation framework based on learning multi-scale crack features. Eng Appl Artificial Intelligence. 2023;126:106876. doi: 10.1016/j.engappai.2023.106876

[2] Ma D, Fang H, Wang N, Zhang C, Dong J, Hu H. Automatic detection and counting system for pavement cracks based on PCGAN and YOLO-MF. IEEE Trans Intell Transport Syst. 2022;23(11):22166–78. doi: 10.1109/tits.2022.3161960

[3] Qu Z, Cao C, Liu L, Zhou D-Y. A Deeply supervised convolutional neural network for pavement crack detection with multiscale feature fusion. IEEE Trans Neural Netw Learn Syst. 2022;33(9):4890–9. doi: 10.1109/TNNLS.2021.3062070 33720835

[4] Xie S, Tu Z. Holistically-nested edge detection. In: 2015 IEEE International Conference on Computer Vision (ICCV), 2015. 1395–403. 10.1109/iccv.2015.164

[5] Al-maqtari O, Peng B, Al-Huda Z, Al-Malahi A, Maqtary N. Lightweight yet effective: a modular approach to crack segmentation. IEEE Trans Intell Veh. 2024;9(12):7961–72. doi: 10.1109/tiv.2024.3405495

[6] Howard A, Sandler M, Chen B, Wang W, Chen L-C, Tan M, et al. Searching for MobileNetV3. In: 2019 IEEE/CVF International Conference on Computer Vision (ICCV), 2019. 1314–24. 10.1109/iccv.2019.00140

[7] Zhang J, Zhang S, Li D, Wang J, Wang J. Crack segmentation network via difference convolution-based encoder and hybrid CNN-Mamba multi-scale attention. Pattern Recognition. 2025;167:111723. doi: 10.1016/j.patcog.2025.111723

[8] Ronneberger O, Fischer P, Brox T. U-net: convolutional networks for biomedical image segmentation. Medical image computing and computer-assisted intervention–MICCAI 2015: 18th international conference, Munich, Germany, October 5-9, 2015, proceedings, part III 18. Springer; 2015. 234–41.

[9] Zhou Z, Siddiquee MMR, Tajbakhsh N, Liang J. UNet++: redesigning skip connections to exploit multiscale features in image segmentation. IEEE Trans Med Imaging. 2020;39(6):1856–67. doi: 10.1109/TMI.2019.2959609 31841402 PMC7357299

[10] Ding X, Huang Y, Tian X, Zhao Y, Zhang Q, Gao Z. MEFA-unet: multi-scale feature extraction and fusion attentional unet for segmenting short process of incus in otologic microsurgical scenarios. Comp Methods Prog Biomed. 2025;109199. 10.1016/j.cmpb.2025.109199 41490517

[11] Guo T, Zhao W, Peng J. Ret-UNet: enhancing medical image segmentation with self-retention. Array. 2026;29:100653. doi: 10.1016/j.array.2025.100653

[12] Jadhav A, Rasool A, Gyanchandani M. Bias corrected twin squeeze-and-excitation attention enhanced UNet for brain tumor segmentation. Biomedical Signal Processing and Control. 2026;115:109390. doi: 10.1016/j.bspc.2025.109390

[13] Wang L, Li R, Zhang C, Fang S, Duan C, Meng X, et al. UNetFormer: a UNet-like transformer for efficient semantic segmentation of remote sensing urban scene imagery. ISPRS J Photogram Remote Sens. 2022;190:196–214. doi: 10.1016/j.isprsjprs.2022.06.008

[14] Vaswani A, Shazeer N, Parmar N, Uszkoreit J, Jones L, Gomez AN. Attention is all you need. Advances in neural information processing systems. 2017;30.

[15] Dosovitskiy A, Beyer L, Kolesnikov A, Weissenborn D, Zhai X, Unterthiner T. An image is worth 16x16 words: transformers for image recognition at scale. arXiv preprint. 2020. doi: 10.48550/arXiv.2010.11929

[16] Deng Y, Ma J, Wu Z, Wang W, Liu H. DSR-Net: distinct selective rollback queries for road cracks detection with detection transformer. Digital Signal Processing. 2025;164:105266. doi: 10.1016/j.dsp.2025.105266

[17] Zhou Q, Qu Z, Ju F. A lightweight network for crack detection with split exchange convolution and multi-scale features fusion. IEEE Trans Intell Veh. 2023;8(3):2296–306. doi: 10.1109/tiv.2022.3210299

[18] Yang F, Zhang L, Yu S, Prokhorov D, Mei X, Ling H. Feature pyramid and hierarchical boosting network for pavement crack detection. IEEE Trans Intell Transport Syst. 2020;21(4):1525–35. doi: 10.1109/tits.2019.2910595

[19] Stricker R, Eisenbach M, Sesselmann M, Debes K, Gross H-M. Improving visual road condition assessment by extensive experiments on the extended GAPs dataset. In: 2019 International Joint Conference on Neural Networks (IJCNN), 2019. 1–8. 10.1109/ijcnn.2019.8852257

[20] Amhaz R, Chambon S, Idier J, Baltazart V. Automatic crack detection on two-dimensional pavement images: an algorithm based on minimal path selection. IEEE Trans Intell Transport Syst. 2016;17(10):2718–29. doi: 10.1109/tits.2015.2477675

[21] Fan L, Li S, Li Y, Li B, Cao D, Wang F-Y. Pavement cracks coupled with shadows: a new shadow-crack dataset and a shadow-removal-oriented crack detection approach. IEEE/CAA J Autom Sinica. 2023;10(7):1593–607. doi: 10.1109/jas.2023.123447

[22] Xu J, Tong L. LB-UNet: a lightweight boundary-assisted UNet for skin lesion segmentation. In: International Conference on Medical Image Computing and Computer-Assisted Intervention, 2024. 361–71.

[23] Huang C, Wu Z, Xi H, Zhu J. kMaXU: medical image segmentation U-Net with k-means Mask Transformer and contrastive cluster assignment. Pattern Recognition. 2025;161:111274. doi: 10.1016/j.patcog.2024.111274

[24] Cao H, Wang Y, Chen J, Jiang D, Zhang X, Tian Q, et al. Swin-unet: Unet-like pure transformer for medical image segmentation. In: European conference on computer vision. Springer; 2022. 205–18.

[25] Yu Y, Zhang Y, Yu J, Yue J. Lightweight decoder U-net crack segmentation network based on depthwise separable convolution. Multimedia Systems. 2024;30(5). doi: 10.1007/s00530-024-01509-3

[26] Zim AH, Iqbal A, Al-Huda Z, Malik A, Kuribayashi M. EfficientCrackNet: a lightweight model for crack segmentation. In: 2025 IEEE/CVF Winter Conference on Applications of Computer Vision (WACV), 2025. 6279–89. 10.1109/wacv61041.2025.00612

[27] Zhang Z, Peng B, Zhao T. An ultra-lightweight network combining Mamba and frequency-domain feature extraction for pavement tiny-crack segmentation. Expert Syst Appl. 2025;264:125941. doi: 10.1016/j.eswa.2024.125941

[28] Chen L, Tang YM, Ng HKC, Yung KL. Multi-channel convolutional neural network and decision-level fusion for multi-part cover fault diagnosis. Eng Struct. 2026;357:122472. doi: 10.1016/j.engstruct.2026.122472

[29] Chen L, Ji M, Tang Y, Ma Y, Wen S, Geda MW. Siamese CNN-BiMGRU integrating multihead attention mechanism and its application to multipart covers fault detection under small-scale samples condition. Struct Health Monitor. 2025. doi: 10.1177/14759217251370360

[30] Bhargavi K, Jyothi S. A survey on threshold based segmentation technique in image processing. Int J Innov Res Develop. 2014;3(12):234–9.

[31] Li P, Wang M, Fan Z, Huang H, Zhu G, Zhuang J. OUR-Net: a multi-frequency network with octave max unpooling and octave convolution residual block for pavement crack segmentation. IEEE Trans Intell Transport Syst. 2024;25(10):13833–48. doi: 10.1109/tits.2024.3405995

[32] Dong J, Wang N, Fang H, Guo W, Li B, Zhai K. MFAFNet: an innovative crack intelligent segmentation method based on multi-layer feature association fusion network. Adv Eng Inform. 2024;62:102584. doi: 10.1016/j.aei.2024.102584

[33] Song Q, Yao W, Tian H, Guo Y, Chandren Muniyandi R, An Y. Two-stage framework with improved U-Net based on self-supervised contrastive learning for pavement crack segmentation. Expert Syst Appl. 2024;238:122406. doi: 10.1016/j.eswa.2023.122406

[34] Wu X, Wang L, Wu C, Guo C, Yan H, Qiao Z. Semantic segmentation of remote sensing images using multiway fusion network. Signal Processing. 2024;215:109272. doi: 10.1016/j.sigpro.2023.109272

[35] Ni M, Chen L, Shi P, Ren R. RepCrack: an efficient pavement crack segmentation method based on structural re-parameterization. Eng Appl Artificial Intelligence. 2025;141:109791. doi: 10.1016/j.engappai.2024.109791

[36] Fan Y, Hu Z, Li Q, Sun Y, Chen J, Zhou Q. CrackNet: a hybrid model for crack segmentation with dynamic loss function. Sensors (Basel). 2024;24(22):7134. doi: 10.3390/s24227134 39598912 PMC11598754

[37] Liu Y, Yao J, Lu X, Xie R, Li L. DeepCrack: a deep hierarchical feature learning architecture for crack segmentation. Neurocomputing. 2019;338:139–53. doi: 10.1016/j.neucom.2019.01.036

[38] Ruan J, Xie M, Gao J, Liu T, Fu Y. Ege-unet: an efficient group enhanced unet for skin lesion segmentation. In: International conference on medical image computing and computer-assisted intervention. Springer; 2023. 481–90.

[39] Tan M, Le Q. Efficientnet: Rethinking model scaling for convolutional neural networks. In: International conference on machine learning. PMLR; 2019. 6105–14.

[40] Ma N, Zhang X, Zheng H-T, Sun J. ShuffleNet V2: practical guidelines for efficient CNN architecture design. Lecture notes in computer science. Springer International Publishing. 2018. 122–38. doi: 10.1007/978-3-030-01264-9_8

